# Supplementary material for: Scoping Review of Yoga in Schools: Mental Health and Cognitive Outcomes in Both Neurotypical and Neurodiverse Youth Populations
Source: Children (Basel). 2022 Jun 8;9(6):849. doi: 10.3390/children9060849 (PMC9222138; doi:10.3390/children9060849)
Supplement: Supplementary file 1 [file children-09-00849-s001.zip › Supplementary File - Search Strategy.pdf]

Scoping review search terms and strategy

Supplementary Table S1: Search terms

Yoga, school, youth and neurodevelopmental terms	Mental health and cognitive terms
Yoga, Yoga-based, Yoga-inspired<br>Mind-body<br>Education<br>Classroom, Class-based<br>School<br>College<br>Adolescent<br>Young people<br>Youth<br>Juvenile<br>Teen<br>Children<br>Junior<br>Student<br>Pupil<br>Typically developing<br>Atypically developing<br>Neurodiverse<br>ADHD<br>Autism<br>Learning difficulties<br>Neurodevelopmental	Cognition<br>Inhibition<br>Working memory<br>Shifting<br>Attention<br>Academic performance<br>IQ<br>Mental health<br>Depression<br>Anxiety<br>Self-esteem<br>Self-concept<br>Psychological wellbeing<br>Subjective wellbeing<br>Resilience

Supplementary Table S2: Scoping review example search strategy (Ovid - PsycINFO)

(yoga* or mind-body* or yoga-based* or yoga-inspired*) AND (school* OR college* OR classroom* OR class-based OR educat* NOT uni*) AND (child* or adolescen* or "young people*" or "young person*" or juvenile* or teen* or junior* or youth* or student* or pupil*) AND ((mental health* or "mental well-being*" or "mental wellness*"or "mental illness*" or depress* or anxiety* or anxi* or self-esteem* or self-concept* or "psychological well-being*" or "psychological wellness*" or "psychological health*" or "subjective well-being*" or "subjective wellness*" or resilience* or cogniti* or "cognitive function*" or "cognitive outcome*" or inhibit* or "working memory*" or shifting* or attention* or academi* or "academic achievement*" or "academic performance*" or "academic attainment*" or grade* or "test score*" or IQ* or intelligence* or "typically developing*" or "typical develop*" or healthy* or atypical* or "atypically developing*" or "atypical develop*" or neurodevelop* or "neurodevelopment impairment*" or "neurodevelopment dis*" or "attention deficit hyperactivity disorder*" or ADHD* or hyperactiv* or "autism spectrum disorder*" or Autis* or spectrum* or ASD* or "learning difficult*")).ti,ab.
